# Irritable bowel syndrome and chronic pelvic pain: a systematic review and meta-analysis

**DOI:** 10.1590/1806-9282.20250758

**Published:** 2025-12-08

**Authors:** João Nogueira, Kamilly Ieda Silva Veigas, Lyvia Maria Rodrigues de Sousa Gomes, Rosy Ane de Jesus Pereira Araújo Barros, Plínio da Cunha Leal, Adalgisa de Sousa Paiva Ferreira

**Affiliations:** 1 Universidade Federal do Maranhão, Department of Medicine – São Luís (MA), Brazil.

## INTRODUCTION

The American College of Obstetricians and Gynecologists defines chronic pelvic pain (CPP) as pain symptoms perceived to originate from pelvic organs/structures, typically lasting more than 6 months^1^. It is often associated with negative cognitive, behavioral, sexual, and emotional consequences as well as with symptoms suggestive of the lower urinary tract, sexual, bowel, pelvic floor, myofascial, or gynecological dysfunctions. It can be divided into three main categories: visceral, neuromusculoskeletal, and psychosocial^1,2^.

Visceral causes of CPP include urological, gynecological, and gastrointestinal (GI) origins, with the latter being caused by irritable bowel syndrome (IBS)^1^. IBS is a chronic, often debilitating, and highly prevalent disorder of gut–brain interaction (previously called functional GI disorders). Furthermore, it is one of the most common diagnoses in women with CPP, occurring in up to 35% of them^3^. However, IBS is frequently underdiagnosed or inadequately treated in women with concurrent CPP^2,4^. The diagnosis can be established using the Rome criteria, which require abdominal pain associated with changes in bowel movement frequency and stool consistency^5^.

The prevalence of IBS among individuals with CPP varies widely, with some studies reporting rates as high as one-third of patients^6^. Several factors are classically associated with this comorbidity, including age, female sex, endometriosis, dyspareunia, dysmenorrhea, a history of pelvic inflammatory disease, depression, anxiety, a history of sexual and/or physical abuse, increased healthcare utilization and financial burden, and reduced quality of life^6,7^.

This systematic review and meta-analysis aimed to evaluate the prevalence and identify potential new associated factors in patients with IBS and CPP over the past two decades.

## METHODS

### Study design

This is a systematic review and meta-analysis conducted according to Preferred Reporting Items for Systematic Reviews and Meta-Analysis (PRISMA), under the ID number CRD42024593935, approved in October 2024, in the Prospective International Registry of Systematic Reviews (Prospero).

The study followed the PICO strategy:

- Population (P): Women with CPP or IBS;
- Intervention (I): Not applicable;
- Comparison (C): Control group without CPP or IBS;
- Outcomes (O): Factors associated with CPP and IBS.

### Eligibility criteria

Original studies that investigated CPP and IBS were included in order to answer the question: "What factors can predict the occurrence of IBS in women with CPP?."

Review articles, duplicate publications, case reports, and studies focusing exclusively on either IBS or CPP were excluded. Studies involving patients with organ failure, obesity, diabetes mellitus, thyroid, collagen, or hematologic disorders, visceral cancer, conditions causing GI symptoms, antibiotic use, gynecological or urological disorders, pregnancy, inguinal hernia, stroke, severe psychotic episodes, intellectual disability, dementia, a history of abdominal surgery, or individuals who were incarcerated were also excluded.

### Sources and search strategy

The literature search was conducted between June 2024 and March 2025 using Scielo, PubMed, and Google Scholar platforms. The descriptors used were "pelvic pain," "irritable bowel syndrome," and "chronic pain," combined with the Boolean operator AND, covering studies from the past 20 years.

Two researchers performed these steps independently, with a third reviewer consulted in cases of disagreement or divergence.

### Data recording

A database was created using Microsoft Excel 2019. The factors analyzed were grouped into sociodemographic characteristics (age, location, marital status, education level, and degree of urbanization), lifestyle and behavioral factors (alcohol consumption, smoking, physical inactivity, and oral contraceptive use), comorbidities and clinical history (previous surgeries, endometriosis, adenomyosis, and hypertension), mental health and psychosocial factors (occupational stress, insomnia, sexual violence, depression, and anxiety), pain and related conditions (abdominal pain, fibromyalgia, migraine, back pain, dysmenorrhea, dyspareunia, and pain duration), and GI signs and symptoms (stool characteristics, constipation, diarrhea, abdominal distension, mucus or blood in stool, gas, and frequency of bowel movement).

### Synthesis results

To integrate the findings of the included studies, the results were summarized qualitatively, and the quantitative data were combined and presented descriptively in tabular form.

For the meta-analysis, studies reporting the number of IBS cases among patients with CPP were included. Data were synthesized using Review Manager (RevMan, Version 5.4), employing the random-effects model. The pooled proportion and the corresponding 95%CIs were calculated and visually presented in a forest plot. Heterogeneity was assessed using the Q statistic (Cochran's Q), Higgins’ I² statistic, where values above 50% were considered indicative of substantial heterogeneity, and the 95% prediction interval. A p<0.05 for the Q statistic was considered statistically significant for heterogeneity.

### Bias analysis

The risk of bias was analyzed using the National Heart, Lung, and Blood Institute^8^ Observational Study Assessment Toolkit.

## RESULTS

### Study selection

**Figure 1:**
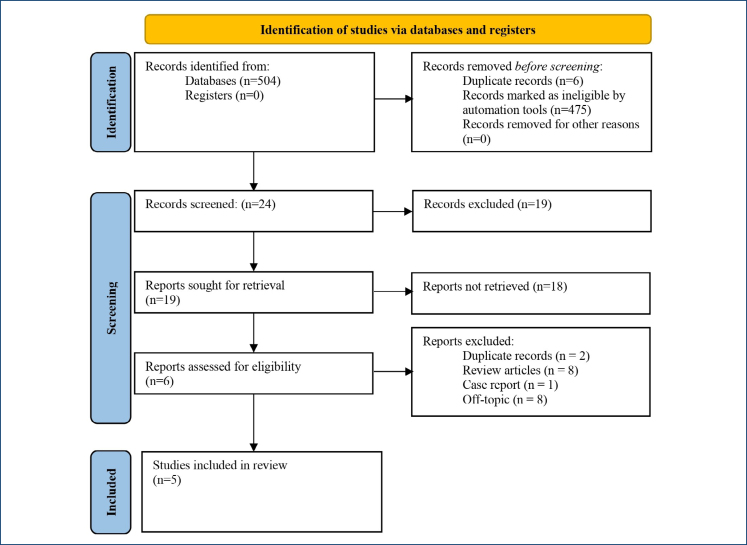
Article selection flowchart.

A total of 504 articles were identified, 24 were selected for further analysis, and 18 studies were excluded due to duplication or not addressing both topics under investigation. As a result, five studies were selected for the systematic review (Figure 1).

### Characterization of studies

In the included studies, two did not specify their population selection methods, while others used systematic sampling, a longitudinal database, or a population-based cohort. In three studies, participants’ ages ranged from 17 to 51 years; two did not report age. In one study, IBS was diagnosed first, followed by an assessment of CPP, whereas in four studies, CPP was the initial diagnosis, with subsequent evaluation of its association with IBS (Table 1).

**Table 1:** Characteristics, prevalence, and associated factors of irritable bowel syndrome in chronic pelvic pain, and risk of bias in the selected studies.

Authors	Year	Diagnostic criteria for IBS	Groups (n)		Subgroups (n)				CPP/IBS prevalence (%)	Prevalence		Associated factors*						Risk of bias
										Psychosocial and health factors	Chronic pain syndromes	Sociodemographic characteristics	Lifestyle and behavioral factors	Comorbidities and clinical history	Mental health and psychosocial factors	Pain and related conditions	Gastrointestinal signs and symptoms	
					CPP		NCPP											
			CPP	NCPP	IBS	NIBS	IBS	NIBS										
Georgescu et al.^9^	2019	ROMA III	20	20	20	–	20	–	NA	65.0–90.0%	–	0.51	0.71	0.01	<0.001	0.003	–	ii
Lessa et al.^10^	2013	ROMA III	235	235	46	189	25	210	19.6	27.1–58.3%	54.2–85.4%	NR	NR	–	0.002	0.002	<0.001	i
Williams et al.^7^	2004	ROMA I	246	–	48	198	–	–	19.5	38.4–41.7%	5.7–37.5%	–	–	0.1	0.7	0.6	–	ii
Tachawiwat and Cheewadhanaraks^12^	2012	ROMA II	987	–	341	646	–	–	34.5	–	37.9–57.4%	–	–	–	–	–	–	i
Choung et al.^11^	2010	ROMA III	67	–	26	41	–	–	38.8	–	–	0.07	0.47	0.05	–	<0.001	–	ii

IBS: irritable bowel syndrome; CPP: chronic pelvic pain; NCPP: no chronic pelvic pain; NIBS: no irritable bowel syndrome;

* Lowest p-value presented for each category; NA: not applicable; NR: not reported; i: Good; ii: Fair.

### Prevalence of irritable bowel syndrome in chronic pelvic pain

The studies were published over 5 years and involved sample sizes ranging from 40 to 987 participants. Four studies compared the presence of IBS and CPP to control groups, though one did not clarify group definitions. The studies were conducted in the United States (2), Romania (1), Brazil (1), and Thailand (1). IBS diagnoses were based on the Rome criteria (I–III). One study^9^ included IBS as an inclusion criterion, preventing prevalence calculation. The other four studies reported IBS prevalence in patients with and without CPP, with rates among those with CPP ranging from 19.5 to 38.8%. One study^10^ also reported an 11.9% IBS prevalence in individuals without CPP (Table 1).

### Factors associated with chronic pelvic pain/irritable bowel syndrome

All five studies evaluated two of the six factors analyzed in this review: psychosocial and mental health factors and chronic pain syndromes in patients with IBS with and without CPP, assessed concurrently. Among individuals with CPP and IBS, the prevalence of mental health conditions ranged from 27.1 to 90.0%; for chronic pain conditions, the prevalence ranged from 5.7 to 85.4% (Table 1).

Sociodemographic factors (urbanization and location) were associated with IBS/CPP comorbidity, while age and marital status were not. Regarding mental health and psychosocial factors, occupational stress and insomnia were not associated with CPP and IBS, whereas a significant association was found between sexual violence and the co-occurrence of IBS and CPP. Lifestyle and behavioral factors, including alcohol consumption, smoking, oral contraceptive use, and physical inactivity, did not demonstrate significant associations. Comorbidities and clinical history were associated with inflammatory markers and endometriosis but not with biochemical results and blood count. Pain and related conditions showed positive associations across all parameters studied, including abdominal pain, fibromyalgia, migraine, back pain, and widespread pain, as did GI symptoms, such as hard stools, constipation, diarrhea, abdominal distension, gas, and the presence of mucus and/or blood in the stool (Table 1).

### Meta-analysis

**Figure 2:**
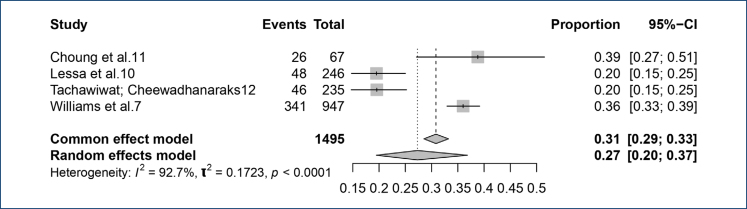
Forest plot of the prevalence of irritable bowel syndrome in patients with chronic pelvic pain.

Four studies reported the number of IBS cases among patients with CPP, allowing for the calculation of a prevalence meta-analysis. In the group with CPP, the mean prevalence of IBS was 28.7%. When adjusted using a random-effects model, the prevalence decreased to 27.0% (Figure 2).

### Risk of bias

The studies showed limitations in the justification sample size (Q7) and control of confounding factors (Q14), and none provided information on adequate follow-up (Q12). Also, the clarity regarding inclusion criteria (Q2) and sampling description (Q5) varied, with some failing to report these aspects. Despite these limitations, most studies adhered to rigorous methodological standards in defining the study population and applying analytical methods, thus contributing to the reliability of the findings (Table 1).

## DISCUSSION

This review identified a pooled prevalence of 27.0% for IBS among patients with CPP, which is slightly lower than previously reported estimates, such as the 35.0% prevalence cited two decades ago. This decline may reflect variations in diagnostic criteria, differences in study populations, or increased awareness and diagnosis of IBS^3,6^.

Age, history of physical or sexual abuse, cystitis, endometriosis, adenomyosis, anxiety, depression, and dyspareunia were reaffirmed as factors associated with patients with IBS and CPP^3,6^. New associations were detected, such as pain duration, low back pain, greater pain somatization, inflammatory markers, DB, fibromyalgia, and migraine. No significant difference was observed between pain intensity levels and IBS prevalence.

The study by Williams et al.^7^ conducted with 987 women with CPP and published two decades ago, investigated 341 patients with IBS diagnosed using Rome I criteria and 646 patients without IBS, reporting a prevalence of 34.5% (95%CI 31.6–37.5) for the IBS/CPP association. In bivariate analyses (p<0.10), characteristics associated with IBS included age, pelvic floor tension myalgia assessed by tenderness to touch, back muscle pain, endometriosis, piriformis syndrome based on a positive thigh rotation test, adenomyosis, history of physical or sexual abuse in adulthood, higher scores on the Beck Depression Inventory, scores on the Symptom Checklist-90, total number of pain sites, pain duration, chronic pain syndrome, and McGill Pain Questionnaire score. In the final reduced multivariable model, IBS remained significantly associated with back muscle pain (OR 5.37; 95%CI 0.98–29.29), age ≥40 years (OR 1.98; 95%CI 1.27–3.11), Beck Depression Inventory score ≥10 (indicating the presence of depression) (OR 1.93; 95%CI 1.24–3.01), top 24% global score on the Symptom Checklist-90 (OR 1.77; 95%CI 1.09–2.86), presence of 6–8 pain sites (OR 1.67; 95%CI 1.01–2.78), and a history of physical abuse in adulthood (OR 1.51; 95%CI 1.01–2.26).

Another population-based study^11^ focusing on this association, conducted approximately 15 years ago with women, found that 20% of the population experienced CPP, and among these, 22% were also diagnosed with IBS. The study highlighted that the group with overlapping IBS and CPP reported significantly higher somatization scores, including symptoms such as depression, migraine, dizziness, and fatigue, compared to those with IBS or CPP alone. These findings suggest that at least a subset of IBS may share a common pathophysiology with CPP and that somatization could be a key factor in understanding this overlap.

Researchers aimed to assess the prevalence of IBS among patients with CPP based on pain intensity, categorized as mild to moderate and severe, compared to controls, using the Visual Analog Scale (VAS) in cases of dysmenorrhea, dyspareunia, and non-menstrual pelvic pain. The sample included 235 patients in both the study and control groups. IBS was diagnosed in 46 individuals (19.6%) in the CPP group and 25 individuals (10.6%) in the control group. Patients with mild-to-moderate CPP (p=0.028) and those with severe CPP (p=0.036) had a significantly higher prevalence of IBS compared to controls. However, there was no significant difference in IBS prevalence between the mild-to-moderate and severe CPP subgroups (p=0.962)^12^.

The population-based survey conducted by Lessa et al. involved 1,470 women and aimed to determine the prevalence of IBS among women with CPP and identify associated characteristics. The dependent variable was the presence of IBS, diagnosed using the Rome III criteria in women with CPP. Independent variables potentially associated with IBS included age, educational level, duration of pain, physical inactivity, migraine, depression, insomnia, low back pain, dysmenorrhea, dyspareunia, history of abuse, and GI symptoms. The prevalence of IBS among women with CPP was 19.5%. Longer pain duration (p=0.03), low back pain (p=0.002), a history of physical or sexual abuse (p=0.002), and GI complaints were more frequent in the IBS and CPP group than in the group without CPP. The findings suggest several shared features between the two conditions, supporting the hypothesis that IBS and CPP may be part of the same syndrome^10^.

The study by Georgescu et al.^9^ is a cross-sectional observational research involving 40 young females diagnosed with IBS according to the Rome III criteria, which characterizes the profile of IBS associated with CPP in young women. Participants were divided into two matched groups based on CPP symptoms, such as cystalgia, urinary urgency, and dyspareunia. The results indicated that inflammatory markers, including high-sensitivity C-reactive protein (hs-CRP) (p=0.0132) and tumor necrosis factor-alpha (TNF-α) (p=0.001), as well as DB (p=0.0001), were more prevalent in the CPP-positive group. Statistically significant differences were also found in the CPP-positive group in terms of more severe scores for abdominal pain (p=0.0025), fibromyalgia (p=0.0001), migraine (p=0.05), and anxiety (p=0.03). Cystalgia showed a positive correlation with abdominal pain (r=0.59; p=0.005) and DB (r=0.44; p=0.04), while dyspareunia was strongly correlated with fibromyalgia (r=0.48; p=0.03) and DB (r=0.48; p=0.02).

A potential limitation in this review is the variation in the definition of IBS, as the included studies, published across different periods, adopted different diagnostic criteria, such as Rome I, II, or III, or relied on diagnoses made by gastroenterology specialists. Additionally, several relevant aspects were not consistently assessed across studies, including pain centralization and a more comprehensive evaluation of behavioral factors, such as the presence of psychiatric diagnoses and levels of stress^13^.

## CONCLUSION

This systematic review and meta-analysis identified a slightly lower prevalence of IBS among women with CPP compared with the past two decades. Also, newly associated factors have emerged, including pain duration and somatization, low back pain, elevated inflammatory markers, DB, fibromyalgia, and migraine. Further large-scale studies with standardized IBS definitions and consistent assessment of subjective variables are necessary.

## Data Availability

The datasets generated and/or analyzed during the current study are available from the corresponding author upon reasonable request.
